# Robot-Assisted Radical Hysterectomy for Cervical Cancer: Review of Surgical and Oncological Outcomes

**DOI:** 10.5402/2011/872434

**Published:** 2011-11-09

**Authors:** Seracchioli Renato, Mabrouk Mohamed, Solfrini Serena, Montanari Giulia, Ferrini Giulia, Giovanardi Giulia, Raimondo Diego, Schiavina Riccardo

**Affiliations:** ^1^Minimally Invasive Gynecological Surgery Unit, S. Orsola-Malpighi Hospital, University of Bologna, 40138 Bologna, Italy; ^2^Department of Obstetrics and Gynecology, Civil Hospital, 41049 Sassuolo, Italy; ^3^Department of Urology, S. Orsola-Malpighi Hospital, University of Bologna, 40138 Bologna, Italy

## Abstract

Robot-assisted procedures are being increasingly incorporated in gynaecologic oncology. Several studies have confirmed the feasibility and safety of robotic radical hysterectomy for selected patients with early-stage cervical cancer. It has been demonstrated that robotic radical hysterectomy offers an advantage over other surgical approaches with regard to operative time, blood loss, and hospital stay. Also initial evidences concerning oncological outcomes seem to confirm the equivalence to traditional open technique. Despite the fact that costs of robotic system are still high, they could be partially offset by several health-related and social benefits: less pain, faster dismissal, and return to full activity than other surgical approaches. The development of robotic technology may facilitate the spread of minimally invasive surgery in gynaecological oncology, overcoming some drawbacks of laparoscopic technique for challenging intervention such as radical hysterectomy. Further studies are needed to evaluate overall and disease-free survival of this technique and associated morbidity after adjuvant therapies.

## 1. Introduction

Radical hysterectomy is considered the standard treatment for patients with early-stage cervical cancer [1]. In the past two decades, the gynaecologic oncologic surgeons performed minimally invasive techniques in order to decrease morbidity while maintaining surgical and oncological outcomes. Many studies showed the safety and feasibility of laparoscopic radical hysterectomy for the treatment of early-stage cervical cancer [2, 3]. The laparoscopic approach provides comparable long-term outcomes to open radical hysterectomy by adding benefits of minimally invasive surgery in terms of blood loss, analgesic requirement, and hospital stay [2]. Despite all these clear advantages, laparoscopic radical hysterectomy was not widely adopted in surgical practice, probably due to some drawbacks of this technique: long learning curve, two-dimensional view, poor ergonomics surgeon position, and limited instruments movements. These conditions negatively influenced the surgical performance, resulting in more tremor, fatigue, and subsequent less accuracy. Robot-assisted technique through the DaVinci Surgical System (Intuitive Surgical Inc., Sunnyvale, Calif, USA) emerged in the contest of minimally invasive surgery to overcome shortcomings of conventional laparoscopy. Robotic system provides three-dimensional view, more ergonomic surgeon position and articulated wrist-like instruments, increasing surgical precision, and dexterity [4, 5]. The robotic application grew rapidly in gynaecological oncology field, especially for technically challenging procedures by laparoscopy, such as radical hysterectomy [6–9].

In the present paper we sought to review the available descriptive and comparative evidences concerning surgical and oncological outcomes of robot-assisted radical hysterectomy for early cervical cancer. 

## 2. Surgical Outcomes

### 2.1. Operative Time and Learning Curve

Longer operative time and learning curve are among the reasons why the minimally invasive staging has not yet been adopted worldwide in gynaecological oncology practice. For robotic system total operative time consists of docking time and console time. The first is the time needed to assemble instruments and attach patient to the robot: advancing the column to the operating table, fastening the robotic arms to the inserted trocars, and introducing the laparoscope. Console time is defined as the surgical time needed to perform the entire operation at the console. Retrospective studies on robotic radical hysterectomy that have considered operative time as surgical outcome reported similar results. Sert and Eraker described 25 patients with early stage cervical cancer who underwent robot-assisted radical hysterectomy and pelvic lymph node dissection, showing a total mean time of 219 min, with a mean console time of 170 min [10]. The multi-institutional study by Lowe et al. on 10 type II and 32 type III robotic radical hysterectomy found a median operating time, from skin incision and skin closure, of 215 minutes [11]. All comparative studies between open (ORH), laparoscopic (TLRH), and robotic radical hysterectomy (RRH) considered the operative time using different definitions. Boggess et al. compared the outcomes of 51 patients who underwent robotically assisted hysterectomy with 41 treated by open type III radical hysterectomy. The operative time, defined as first skin incision to skin closure, was significantly shorter for RRH than ORH (210.9 ± 45.5 min versus 247.8 ± 48.8 min) [12]. Nehzat et al. prospectively analyzed that 30 patients underwent TLRH for cervical cancer and 13 patients underwent RRH with no statistical differences regarding mean operative time (323 min versus 318 min) [13]. The prospective study by Magrina et al. compared 27 patients who underwent robotic radical or modified radical hysterectomy with laparoscopic and laparotomic approach. They found a similar operative time from skin incision to skin closure, between robotic (189.6 min) and laparotomic radical hysterectomy, but it was significantly shorter than laparoscopic approach. In particular, this result was retrieved in the subgroup of patients who underwent the modified radical hysterectomy but not the radical one. The mean console time was 150.4 minutes, and it was longer for the radical (182.1 min) than the modified radical hysterectomy (126.1 min) [14]. Moreover, Lambaudie et al. confirmed a significant difference in operative time between three approaches, showing a longer time for the laparoscopic procedure [7]. On the contrary, Estape et al. compared 32 radical hysterectomy by robotic approach with 17 by laparoscopy and 14 by open surgery, showing no significant difference for the operative time, defined from the insertion of the foley catheter and the closing of the last trocar site [15].

The lack of standardization in “operating time” definitions makes more difficult to draw comprehensive conclusions. From previous studies cited it can be affirmed that robotic and open technique seem to have a similar mean operating time, which is significantly shorter than conventional laparoscopy. This issue suggests that robotic technique may simplify and accelerate some demanding steps of laparoscopic radical hysterectomy. Overall, surgical experience and personal learning curve may influence the length of operative time. 

While a learning curve has been extensively described for laparoscopic surgery, little is known about the use of robotic platform in gynaecological oncology. To date no comparative studies evaluated the learning curve for robotic and laparoscopic radical hysterectomy. There are several parameters to be estimated which are able to influence this outcome for robotic system. Firstly, it must be considered the time required to prepare and activate the robot, the time to complete the operation, and finally the number of cases necessary to stabilize surgeon operative time. Additional time for robotic system preparation is considered a disadvantage. In the literature is reported a docking time until 68 minutes, defined from patients entry in the operating room to onset of surgery [16]. Several studies demonstrated a significant decrease of robotic docking time as the surgeon and assistant gained experience, The reported mean docking time was 10 min at the beginning and 2-3 minutes at the end of the learning curve [13–17]. In conclusion, docking time seems to have an influence in overall operative time only in training phase, because expertise is quickly gained for robotic setup. 

The operative learning curve seems to be shorter for robotic than for conventional laparoscopy [18, 19]. Fanning et al. performed 20 robotic radical hysterectomies showing a median operative time of 6.5 hours with a reducing time of surgery from 8 hours to 3.5 hours after 20 procedures [17]. Boggess et al. in a subanalysis of RRH found a decrease of 50 minutes between the first (243.4 min) and the ultimate 12 patients (193.2 min) [12]. Furthermore, Lenihan et al. indicated that after 50 cases the surgeons developed a standard technique, stabilizing operative time and improving outcomes for benign gynaecological robotic surgery [18].

In conclusion, technical advantages of robotic system may allow to perform advanced surgical procedures, such as radical hysterectomy, with a faster learning curve than conventional laparoscopy. Unfortunately, to date no consensus and standardisation are reached for the number of cases required to obtain or maintain robotic practice by one institution. Training programs and credentialing guidelines about robotic special skills and proficiency are ongoing. 

### 2.2. Blood Loss and Blood Transfusion

There is general agreement about the significant decrease of intraoperative bleeding in minimally invasive surgery. This benefit is confirmed also for robotic-assisted technique. The literature reported similar values of blood loss comparing robotic with laparoscopic radical hysterectomy, with important differences with respect to open surgery.  Table 1 summarizes the estimate blood loss for robotic, laparoscopic, and laparotomy techniques for radical hysterectomy. In patients who underwent robotic or laparoscopy radical hysterectomy, the overall rate of blood transfusions was very low. Blood transfusion rates for symptomatic postoperative anaemia after robotic radical hysterectomy vary from 5 to 35% [12, 14, 15, 20, 21]. 

## 3. Morbidity of Robotic-Assisted Radical Hysterectomy

### 3.1. Intraoperative Complications

Minimally invasive surgery provides a lower intraoperative complications rate than open approach, due to a more accurate tissue manipulation and a better anatomic visualization. Robotic surgery may further reduce intraoperative morbidity and improve surgical precision as a consequence of several technical advantages over conventional laparoscopy. Urinary injuries, which may happen during ureterolysis and bladder isolation steps, are frequent reported complications for radical hysterectomy. Sert and Eraker described, among 25 robotic radical hysterectomies, three cases of bladder perforation, which were successfully repaired robotically [10]. The multi-institutional experience by Lowe et al. reported one bladder injury adjacent to the trigone and one ureteral injury [11]. A recent review comparing robotic versus total laparoscopic radical hysterectomy for early cervical cancer found a similar percentage of overall major intraoperative complications rate, about 6%, with a lower rate of vascular and bladder injuries for RRH [2]. Estape et al. reported one cystotomy in the robotic group in a patient with three caesarean sections, and two cystotomies in the laparoscopic group [15]. On the contrary, Nezhat et al. did not note significant differences between robotic and laparoscopic approach with respect to intraoperative complications: in both groups two incidental cystotomies were described [13]. Ko et al. compared 32 cases of ORH to 16 cases RRH: no intraoperative complications occurred in the RRH group, while they reported in the ORH group one case of ureteral transection requiring surgical repair [21]. 

Regarding neural damage, Maggioni et al. reported one obturator nerve injury with temporary mild palsy [20]. Persson et al. found six genitofemoral nerve injuries and one partial obturator nerve palsy at one year of followup in robot-assisted radical hysterectomy with pelvic lymphadenectomy [16]. Recently a higher nerve injuries rate was found for RRH than LRH, probably due to thermal cauterisation [2]. Concerning conversion to laparotomy in RRH, it has been reported only one case with a conversion rate of 2.8% among all 42 patients [11].

### 3.2. Postoperative Complications

There is a general agreement about the substantial advantages of minimally invasive surgery with respect to open technique also in terms of postoperative complications. Comparing robotic or laparoscopic approach to open for radical hysterectomy revealed a lower rate of serious complications for minimally invasive surgery [7, 12, 17]. Otherwise, comparing laparoscopic and robotic approaches, it is still unclear which one is associated with the lower postoperative morbidity. A recent review of the literature described a significant difference in number of major postoperative complications between RRH (9.6%) and TLRH (5.5%). In the RRH group were included 11 cases of vaginal dehiscence, 10 cases of vaginal cuff abscess, and 5 cases of port site hernia [2]. Conversely, Estape et al. demonstrated that the incidence of postoperative complications was less in the robotic group (18.8%) than either the laparoscopic group (23.5%) or the laparotomy group (28.6%). In the first group, one patient developed a pelvic abscess and another one a vaginal evisceration [15]. Two other studies did not observe any significant differences in postoperative outcomes [13, 14]. Magrina et al. described similar early (<6 weeks) major postoperative complications in RRH (7%), LTRH (6%), and ORH (9%) groups [14]. Overall postoperative complications rate described by Lowe et al. was 12%, including deep venous thrombosis (2.4%), pyelonephritis (2.4%), prolonged bladder catheterization of 21 days (2.4%), and infection (4.8%) [11].

There are evidences of an increased relative risk of vaginal cuff complications for minimally invasive hysterectomy techniques when compared to vaginal or abdominal ones [24, 25]. The literature concerning this topic is controversial, also for the lack of a standardized method of classification and registration of postoperative injuries. A recent review showed a higher rate of vaginal dehiscence in the RRH group than in the laparoscopic one [2]. It may be associated with an extensive use of monopolar and bipolar electrosurgery, which may increase thermal damage and devascularisation of the cuff site. Other studies are in disagreement with the prior review and described a similar incidence of vaginal cuff separation comparing laparoscopy and robotic [24, 26]. Moreover, the individual surgical technique may influence the vaginal cuff injury rate. Persson et al. reported also a leaking of lymphatic fluid through vagina and/or vaginal cuff dehiscence in 10 out of 80 women (12%) who underwent robotic radical hysterectomy with pelvic lymphadenectomy. The frequency of this complication was significantly different between three surgeons who performed robotic radical hysterectomy [16].

Table 2 summarizes the number of intra- and postoperative complications in different studies concerning robotic radical hysterectomy. Due to the acceptable overall complication rate, robotic technique is considered a safe and feasible alternative to open approach for patients requiring radical hysterectomy. Moreover, robot-assisted surgery demonstrated some advantages over traditional laparoscopy, causing less vascular and urethral injuries. A recent study by Magrina et al. described the nerve-sparing technique in robot-assisted radical hysterectomy. Technical aspects of nerve-sparing operation can be easily included in the robotic surgery, without compromising surgical radicality. Indeed, nerve-sparing approach improves visualization of the pelvic autonomic nerves facilitating their preservation, reducing bladder and rectal dysfunctions [27].

## 4. Hospital Stay and Costs

The shorter length of hospital stay is one of the most important advantages of minimally invasive surgery. All comparative studies concerning robotic radical hysterectomy reported a mean length of hospital stay of 1-2 days, similar to the laparoscopic group, but significantly shorter than open group [10–12, 14, 15, 21]. Accordingly, robotic surgery provides other advantages, such as lower perioperative complications and reintervention rates, less postoperative pain, and analgesic consumption. All these issues positively influence hospital stay, quality of life, and time to return to full activities, providing a benefit from a medical and socioeconomic point of view. There are no studies about cost-benefit analysis to evaluate whether the robot imposes financial gain or damages to the healthcare system. This subject is controversial; in fact, being a new technology, the expense of the robotic system is still high. Maintenance and materials are more expensive than for conventional laparoscopy. However total costs seem to decrease with the improvement of surgeon experience [28]. High surgical costs may be balanced by a shorter operative time and hospital stay. Moreover, the lower complications rate for complex and high morbidity interventions may increase the cost effectiveness of robotic technique.

## 5. Oncological Outcomes

The primary endpoint to be considered when comparing minimally invasive techniques and conventional laparotomy for gynaecological oncology is the equivalence in terms of surgical staging completeness and survival. Oncological outcomes after radical hysterectomy for early cervical cancer are the number of lymph node retrieved and the recurrence rate. There are controversial results concerning the number of lymph nodes collected by different surgical approaches. Several comparative studies found no significant differences in the number of lymph nodes retrieved between robotic, laparoscopic, and open techniques [14, 21, 23]. A case control study which compared robotic to open type III radical hysterectomy found a nodes retrieval in favour of robotic approach [12]. On the contrary, Maggioni et al. showed a reduced number of lymph node in RRH group compared to the ORH one [20]. More recently, Estape et al. in a case-matched comparative analysis between laparoscopic, robotic, and abdominal radical hysterectomy with lymphadenectomy reported a significantly higher number of nodes for robotic approach and an equivalent rate of positive surgical margins [15]. The increased lymph node yield may be explained by the agility of the robotic instruments that would allow a more comprehensive and accurate node dissection. The number of lymph nodes obtained may further increase following a learning curve.

Although most of the current published series have a short followup and a retrospective design, recurrence rate seems to be equivalent between RRH and TLRH in patients with early cervical cancer [2, 12]. Nezhat et al. reported no recurrences in TLRH and RRH groups at 12 months and in TLRH group at 29 months [13]. In the prospective study by Magrina et al. all patients of the three groups are alive and free from disease at mean followup of 31.1 months [14]. Finally, Cantrell et al. assessed the progression-free and overall survival for 71 women who attempted RRH for cervical cancer. Their experience demonstrated that RRH appears to have equivalent oncological outcomes compared with laparotomic surgery in the first three years of followup. They showed a 94% of progression-free and overall survival in the robotic cohort at 36 months [22].

In conclusion, early data seem to confirm similar oncological outcomes for robotic radical hysterectomy with respect to other surgical modalities in terms of lymph node retrieval and recurrence rate. A prospective randomised trial is currently being performed to test the equivalence for minimally invasive radical hysterectomy (laparoscopy or robotics) over laparotomy in terms of disease-free survival [29].

## 6. Conclusion

Current evidences demonstrated the safety and feasibility of robot-assisted radical hysterectomy for early cervical cancer. Robotic approach provides clear benefits of minimally invasive surgery, such as a reduced blood loss, a lower morbidity, and a faster recovery than open surgery. Although surgical outcomes are similar or slightly improved when compared to laparoscopy, there are multiple potential advantages. In fact, robotic technology may increase surgical precision and reduce operative time and training for technically challenging steps, encouraging the widespread of minimally invasive approach. We have a preliminary experience in robotic-assisted laparoscopy in surgical treatment of benign pathology and of endometrial cancer. Early cervical cancers were usually treated in our centre by radical hysterectomy via conventional laparoscopy, with good results in terms of operative time, functional and oncological outcomes. According to the literature evidence reviewed, robotic radical hysterectomy seems to be a surgical procedure which may benefit from Da Vinci use. Prospective randomized controlled trials will give more definite results, especially concerning surgical outcomes comparing robotic and laparoscopy techniques. Although early data support the equivalence of minimally invasive approach with respect to other modalities in terms of staging completeness, progression-free, and overall survival, further studies with longer followup are needed to confirm survival outcomes and to analyse morbidity after surgery and adjuvant therapies.

## Figures and Tables

**Table 1:** Intraoperative blood loss of robot radical hysterectomy (RRH), laparoscopic radical hysterectomy (TLRH), and open radical hysterectomy (ORH).

	Intraoperative blood loss (mL)		
	RRH	TLRH	ORH
Fanning et al. [17]	300 (100–475)		
Sert and Eraker [10]	57 (10–300)		
Lowe et al. [11]	50 (25–150)		
Persson et al. [16]	150 (25–1300)		
Ko et al. [21]	81.9		665.6
Nezhat et al. [13]	157 (50–400)		200 (100–500)
Boggess et al. [12]	96.5 ± 85.8		416.8 ± 188.1
Maggioni et al. [20]	78		221.8
Cantrell et al. [22]	50 (20–400)		400 (100–1200)
Geisler et al. [23]	165		323
Estape et al. [15]	130 ± 119.4	209.4 ± 169.9	621.4 ± 294.0
Magrina et al. [14]	174.6 (151.1)	254.3 (140.9)	570.3 (220.8)

**Table 2:** Overall intraoperative and postoperative complications of robot radical hysterectomy (RRH), laparoscopic radical hysterectomy (TLRH), and open radical hysterectomy (ORH).

	Patients (*n*)	Intraoperative complications (*n*)	Postoperative complications (*n*)
Kim et al. [9]	10 RRH	0	1
Lowe et al. [11]	42 RRH	2	5
Persson et al. [16]	80 RRH	/	39
Fanning et al. [17]	20 RRH	1	1
Sert and Eraker [10]	25 RRH	4	/
Ko et al. [21]	10 RRH	0	3
	32 ORH	1	7
Boggess et al. [12]	51 RRH	0	4
	49 ORH	2	6
Maggioni et al. [20]	40 RRH	2	19
	40 ORH	5	36
Cantrell et al. [22]	27 RRH	1	2
	64 ORH	1	3
Nezhat et al. [13]	13 RRH	2	4
	30 TLRH	2	6
	27 RRH	0	7
Magrina et al. [14]	31 TLRH	1	5
	35 ORH	2	6
	32 RRH	1	6
Estape et al. [15]	17 TLRH	2	4
	141 ORH	0	4
