# Erratum to “Robot-Assisted Radical Hysterectomy for Cervical Cancer: Review of Surgical and Oncological Outcomes”

**DOI:** 10.1155/2013/305175

**Published:** 2013-01-10

**Authors:** Renato Seracchioli, Mohamed Mabrouk, Serena Solfrini, Giulia Montanari, Giulia Ferrini, Giulia Giovanardi, Diego Raimondo, Riccardo Schiavina

**Affiliations:** ^1^Minimally Invasive Gynecological Surgery Unit, S. Orsola-Malpighi Hospital, Bologna University, 40138 Bologna, Italy; ^2^Departement of Obstetrics and Gynecology, Civil Hospital, 41049 Sassuolo, Italy; ^3^Department of Urology, S. Orsola-Malpighi Hospital, University of Bologna, 40138 Bologna, Italy

In the paper titled “Robot-Assisted Radical Hysterectomy for Cervical Cancer: Review of Surgical and Oncological Outcomes”, all the authors' names and surnames are in reverse order. The authors list should be corrected as follows: Renato Seracchioli, Mohamed Mabrouk, Serena Solfrini, Giulia Montanari, Giulia Ferrini, Giulia Giovanardi, Diego Raimondo, and Riccardo Schiavina.

